# Low Serum Interleukin-10 Is an Independent Predictive Factor for the Risk of Second Event in Clinically Isolated Syndromes

**DOI:** 10.3389/fneur.2019.00604

**Published:** 2019-06-11

**Authors:** Yuzhen Wei, Haoxiao Chang, Hao Feng, Xindi Li, Xinghu Zhang, Linlin Yin

**Affiliations:** ^1^Department of Neurology, Beijing Tiantan Hospital, Capital Medical University, Beijing, China; ^2^China National Clinical Research Center for Neurological Diseases, Beijing, China

**Keywords:** interleukin-10, clinically isolated syndrome, biomarker, relapse, multiple sclerosis, neuromyelitis optica spectrum disorder, progression, conversion

## Abstract

**Objective:** To evaluated the prognostic ability of several serum cytokines in clinically isolated syndrome (CIS) patients regarding second events and conversion to multiple sclerosis (MS) or neuromyelitis optica spectrum disorder (NMOSD).

**Methods:** We enrolled 69 CIS patients whose serum samples were collected during the acute phase of the first onset before immunotherapy. Fifteen other non-inflammatory neurological disorder (OND) patients were also included. The serum levels of interleukin (IL)-2, IL-4, IL-6, IL-10, IL-13, IL-17A, IL-21, IL-23, interferon-γ (IFN-γ), and transforming growth factor beta 1 (TGF-β1) were measured using the human cytokine multiplex assay or ELISA. Patients were seen every 3–6 months. Unscheduled visits occur in case of exacerbations. Clinical measures of disease progression were recorded.

**Results:** Twenty CIS cases had second events during follow-up at a mean time of 15.3 ± 9.9 months. Serum IL-10 levels were significantly lower in CIS patients who relapsed compared to patients who did not. Low serum IL-10 levels were associated with higher risk and shorter times to second events. In clinical correlations, a significantly higher CSF white blood cells count, number of T2 lesions, and gadolinium-enhancing (Gd+) lesions in baseline MRI were found in the low serum IL-10 level group. Of the 20 relapsed cases, seven converted to MS, and eight converted to NMOSD. No significant differences were found in any cytokine levels between these patients at first onset.

**Conclusions:** These findings support using serum IL-10 as a biomarker associated with the risk of relapse and the time to second events in patients with CIS. However, serum cytokine levels can not differentiate between the conversion from CIS to MS or NMOSD.

## Introduction

Idiopathic inflammatory-demyelinating diseases (IIDDs) are monophasic, multiphasic, or progressive disorders characterized by inflammatory demyelination affecting attacks different sites of the central nervous system (CNS) (1). This spectrum represent a broad spectrum of CNS disorders that can be differentiated on the basis of severity, clinical course, lesion distribution, and imaging, laboratory, and pathological findings (1).

Clinically isolated syndrome (CIS) is a term widely used in contemporary neurological practice to describe the first clinical episode in which a patient has symptoms and signs suggestive of an inflammatory demyelinating disorder of the CNS (2). The term “CIS” usually suggests the possibility of multiple sclerosis (MS). However, there can be a considerable overlap between some IIDDs, especially in the initial demyelinating event (IDE). Some CIS will convert to other IIDDs like neuromyelitis optica spectrum disorder (NMOSD), recurrent optic neuritis (ON), and relapsing myelitis during follow-up. More relapses are always associated with poorer recovery of neurological function. The treatment for NMOSD differs from that of MS (3–5). Therefore, early identification of patients at high risk of relapsing activity and prediction of their conversion is crucial for timely treatment escalation or induction therapy (6). Nevertheless, the majority of studies on CIS relapse have focused on its conversion to MS (7). Limited information has been published concerning the evaluation of clinical risk factors for relapse in other multiphasic IIDDs. In addition, very few studies have analyzed the predictive effect of serum cytokine levels on clinical progression (6). A previous study reported the interleukin-8 (IL-8) CSF contents could predict conversion to MS after CIS, suggesting that evaluation of the cytokines in CIS patients may provide useful information for predicting subsequent attacks (6). In the present study, we detected several cytokines in the serum of CIS patients and evaluated their ability to predict second events and conversion from CIS to MS or NMOSD. The associations between serum cytokines levels and clinical findings were also investigated.

## Materials and Methods

### Patients

Between January 2015 and August 2017, 228 consecutive patients hospitalized in the neurology department of Beijing Tiantan Hospital were diagnosed with CIS. Patients meeting the following conditions were included: (1) the serum samples were collected during the acute phase (< 2 weeks from symptom onset) of the first onset and before any immunomodulatory or immunosuppressive therapy, (2) any infectious or other autoimmune comorbidities did not coexist at the time of sample collection, and (3) patients did not receive immunomodulatory treatment before relapse, or during follow-up time for patients without relapse.

Baseline clinical data including gender, age, routine CSF information (white blood cells [WBC] count, protein level, IgG index, oligoclonal band), MRI information (number of T2 lesions, number of gadolinium-enhancing [Gd+] lesions), and the Expanded Disability Status Scale (EDSS) disability score were recorded. Patients were seen every 3–6 months. Unscheduled visits occurred in case of exacerbations. The EDSS score during each clinic visit and the time to second event were prospectively collected. Relapses were defined as the development of new or recurrent neurological symptoms not associated with fever or infection lasting for at least 24 h. NMOSD and MS diagnoses were made according to the 2015 Revised International Criteria (8) and 2010 McDonald's Diagnostic Criteria (9), respectively.

Fifteen patients with other non-inflammatory neurological diseases (OND) were also enrolled (13 women and two men with a mean age of 40.2 years). The OND group included benign intracranial hypertension (*n* = 3), cluster headache (*n* = 3), psychogenic movement disorders (*n* = 3), normal pressure hydrocephalus (*n* = 2), benign paroxysmal positional vertigo (*n* = 2), sleep disturbance (*n* = 1), and vitamin B12 deficiency (*n* = 1).

The study was approved by the Ethics Committee of Beijing Tiantan Hospital Affiliated to Capital Medical University, Beijing, People's Republic of China (No. KY2015-031-02), and written informed consent was obtained from all participants.

### Biomarker Measurement

The CSF samples were centrifuged and the supernatants were collected and stored at −80°C until analyzed. Samples were tested for the aquaporin-4 antibody (AQP4-Ab) using a cell-based assay (CBA) with live HEK-293 cells transiently transfected with full-length M23-AQP4, as described previously (10). The samples were simultaneously assayed using the MILLIPLEX® MAP Human High Sensitivity Cytokine/Chemokine Panels (Cat. Nos. HCYTOMAG-60K, HCYP2M AG-62K; Merck KGaA, Darmstadt, Germany) for the following nine cytokines: IL-2, IL-4, IL-6, IL-10, IL-13, IL-17A, IL-21, IL-23, and IFN-γ. In addition, the levels of TGF-β1 were measured by ELISA (EHC107b.96, NeoBioscience, Shenzhen, China). All samples were assayed in duplicate and all testing was performed according to the manufacturer's protocols and in a manner that was blinded to diagnoses or clinical presentations.

### Statistical Analysis

Statistical analysis was conducted using SPSS 22.0 (International Business Machines Corporation, Chicago, IL, USA). Data were presented as the mean ± SD. Continuous data were compared with the non-parametric Mann–Whitney test. Categorical data were compared with the chi-square or Fisher's exact test. Receiver operating characteristic (ROC) curve analyses were used to determine the best cut-off value based on serum IL-10 levels and CSF WBC count. Univariate and multivariable Cox proportional hazard regression models were used to evaluate the association between serum IL-10 levels and the time to relapse, including the following as covariates: sex and age at the time of the initial demyelinating event, serum IL-10 levels, CSF WBC count, the presence of oligoclonal bands, number of T2 lesions on the initial brain and spinal cord MRI, presence of Gd+ lesions, and EDSS during the first attacks. Dependent variables were first tested in a univariate analysis and included in a multivariate model at a threshold of 0.2 (Wald test) when found to be significant. The time from the initial demyelinating event to relapse was estimated by Kaplan–Meier survival analyses. Log-rank tests were used to compare survival data between low and high serum IL-10 levels. A two-tailed Spearman's rank correlation coefficient was used to ascertain the associations. The significance level was established at *p* < 0.05.

## Results

### Patient Characteristics

**Figure 1:**
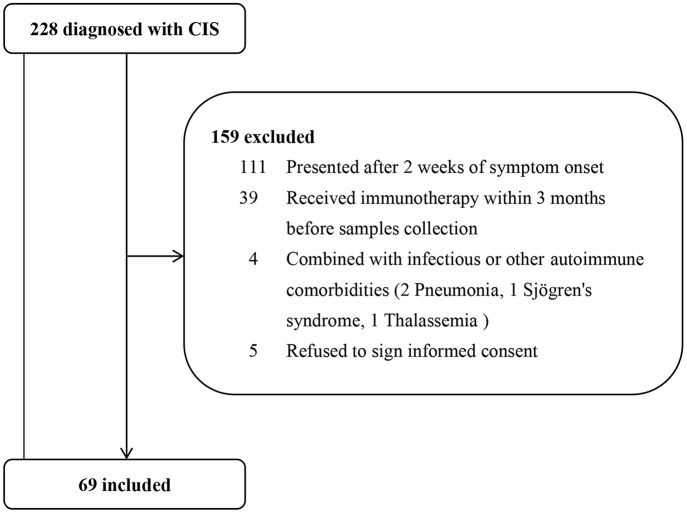
Patient screening process. From Jan. 2015 to Aug. 2017, 228 consecutive patients with clinically isolated syndromes (CIS) were hospitalized in our department. After excluding 159 patients who did not meet the inclusion criteria, we finally included 69 patients.

A total of 69 patients were included in the study. The patient screening process is shown in Figure 1. The average age at first-onset was 41.6 ± 14.7 years. The majority of patients had a monoregional onset (*n* = 46, 66.7%). Of all 69 patients, the affected regions were as follows: optic nerve, 16 (23.2%) patients; brainstem, 21 (30.4%) patients; spinal cord, 43 (62.3%) patients; and cerebrum, 20 (29.0%) patients. No patients were positive for AQP4-Ab in serum when the first attacks occurred. All patients received corticosteroids during acute relapse after serum samples were collected. The mean follow-up time of patients with CIS was 23.3 ± 8.2 months. The median follow-up time was 23.1 (9.5–39.9) months. There was at least 1 year of follow-up for 91.3% (*n* = 63) of patients and 2 years for 46.4% (*n* = 32). Of 69 patients, 20 (29.0%) had second events during follow-up at a mean time of 15.3 ± 9.9 months. Characteristics of CIS patients who relapsed during follow-up are presented in Supplementary Table S1. The baseline characteristics of patient with relapse (R) and non-relapse (NR) during follow-up are detailed in Table 1. CIS-R did not differ from CIS-NR in any analyzed parameters, except for differences in the CSF WBC count (13.3 ± 14.3 vs. 9.7 ± 16.8, *p* = 0.041). Of 20 CIS-R patients, seven converted to MS according to the 2010 McDonald's Diagnostic Criteria; eight converted to NMOSD according to the 2015 Revised International Criteria; three patients were diagnosed with relapsing myelitis; one with relapsing brainstem encephalitis; and one with tumefactive demyelination. Of the eight NMOSD patients, only one patient was positive for serum AQP4-Ab at relapse.

**Table 1:** Characteristics of the patients with clinically isolated syndromes (CIS).

**Clinical characteristics**	**CIS-NR (*n* = 49)**	**CIS-R (*n* = 20)**	***p*-value**
Sex ratio (F/M)	31/18	13/7	0.892
Age, years, mean (SD)	41.7 (14.2)	41.2 (16.1)	0.895
**Onset location, n (%)**			
Monoregional	33 (67.3)	13 (65.0)	0.851
Polyregional	16 (32.7)	7 (35.0)	0.851
**Onset location, n (%)**			
Optic nerve	14 (28.6)	2 (10.0)	0.179
Spinal cord	29 (59.2)	14 (70.0)	0.400
Cerebrum	13 (26.5)	7 (35.0)	0.482
Brainstem	14 (28.6)	7 (35.0)	0.599
EDSS, mean (SD)	3.9 (1.5)	4.4 (1.5)	0.225
**CSF analysis at attack**			
CSF WBC count, mean (SD), /ul	9.7 (16.8)	13.3 (14.3)	0.041^*^
CSF protein level, mean (SD), mg/dl	35.6 (19.7)	37.3 (20.8)	0.672
CSF IgG index, mean (SD)	1.0 (1.5)	0.8 (1.0)	0.197
Positive oligoclonal bands, n (%)	24 (49.0)	13 (65.0)	0.226
Follow-up time, mean (SD), months	22.4 (7.9)	25.7 (8.5)	0.111

Continuous variables were shown as the mean (SD), and categorical variables were described as percentages. CIS-NR, CIS patients who did not relapse during follow-up; CIS-R, CIS patients who relapsed during follow-up; F, female; M, male; EDSS, Expanded Disability Status Scale; CSF, cerebrospinal fluid; WBC, white blood cell;

* *p < 0.05*.

### Serum IL-10 Levels Are Lower in CIS Patients Who Relapse During Follow-Up

**Figure 2:**
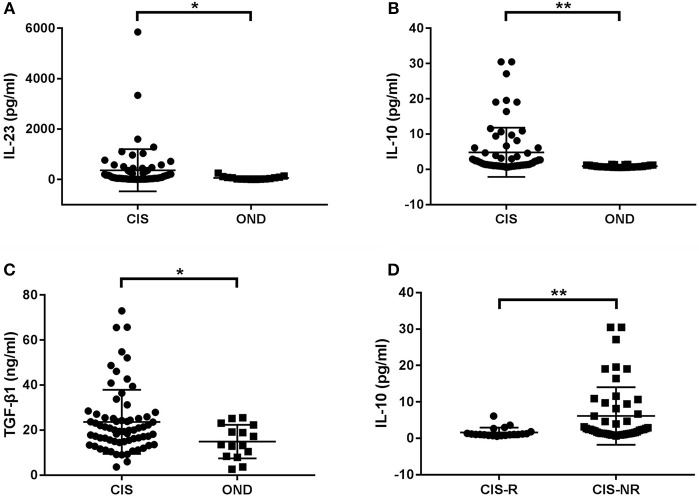
Cytokine levels in serum of patients with clinically isolated syndrome (CIS) and other non-inflammatory neurological diseases (OND). **(A)** Patients with CIS had significantly higher levels of serum IL-23 than those with OND (*p* = 0.015). **(B)** The serum IL-10 levels in CIS were significantly higher than in OND (*p* < 0.001). **(C)** The serum TGF-β1 levels in CIS were significantly higher than in OND (*p* = 0.022). **(D)** The serum IL-10 levels in CIS patients who relapsed (CIS-R) were significantly lower than in patients who did not relapse (CIS-NR) during follow-up (*p* = 0.001). *p < 0.05 and **p < 0.01 represent statistical significance using the Mann-Whitney *U*-test.

First, we compared serum cytokines levels between the entire cohort of CIS patients and control subjects with OND. The IL-23, IL-10, and TGF-β1 levels were significantly higher in patients with CIS than in patients with OND (*p* = 0.015, *p* < 0.001, and *p* = 0.022, respectively; Figures 2A–C). No significant differences were found in IL-2, IL-4, IL-6, IL-13, IL-17A, IL-21, and IFN-γ levels between CIS and OND patients.

Further stratification of the CIS group revealed that only serum IL-10 levels were significantly lower in patients with CIS who relapsed during follow-up compared to patients who did not (*p* = 0.001; Figure 2D).

### Low Serum IL-10 Levels Are a Predictor of Relapse for CIS Patients

**Figure 3:**
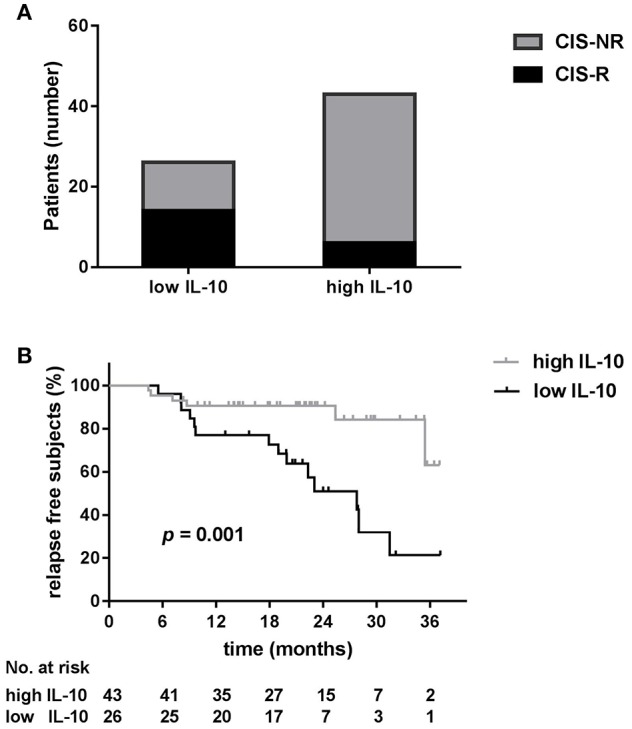
Low serum IL-10 levels are a predictor of relapse for clinically isolated syndrome (CIS) patients. **(A)** There was a significantly higher proportion of patients with clinical relapse (R) during follow-up among low IL-10 (<1.3 pg/ml) CIS patients than among high IL-10 (≥1.3 pg/ml) CIS patients (70.0 vs. 30.0%, *p* < 0.001). **(B)** Kaplan–Meier curves show that the time to first relapse was shorter in CIS patients with low serum levels of IL-10 (log-rank, *p* = 0.001).

ROC analysis showed that the areas under the ROC curve (AUC) for serum IL-10 were significant for relapse (AUC = 0.77; 95% confidence interval[CI] 0.65–0.89; *p* = 0.001; Supplementary Figure 1A). An IL-10 value of 1.3 pg/ml was determined to be the optimal cut-off point for predicting patient relapse. The sensitivity and specificity of this cut-off were 0.700 and 0.755, respectively and a total of 26 CIS patients (37.7%) had IL-10 levels below the cut-off point. The risk of relapse was higher in patients with lower serum IL-10 levels. There was a significantly higher proportion of patients with clinical relapses during follow-up among the low IL-10 group (IL-10 < 1.3 pg/ml) than among the high IL-10 group (IL-10 ≥ 1.3 pg/ml) (70.0 vs. 30.0%, *p* < 0.001; Figure 3A).

The CSF WBC cut-off value was also determined by ROC analysis to be 5.5/ul (AUC = 0.66, 95% CI 0.51–0.81, *p* = 0.042; Supplementary Figure 1B).

Table 2 shows the results of the univariate and multivariable Cox regression models throughout the entire follow-up. In the univariate analysis, serum IL-10 < 1.3 pg/ml (hazard ratio [HR] = 4.36, *p* = 0.003), CSF WBC count >5.5 /ul (HR = 3.80, *p* = 0.010), and Gd+ lesions presence in baseline MRI (HR = 8.60, *p* = 0.036) were associated with an increased risk of relapse. In the multivariate analysis, only the serum IL-10 < 1.3 pg/ml (HR = 2.85, *p* = 0.045) was predictive of relapse.

**Table 2:** Cox regression models containing univariate and multivariate analyses of factors related to the time to relapse.

**Variable**	**Univariate analysis HR (95% CI)**	***p***	**Multivariate analysis HR (95% CI)**	***p***
Sex	1.01 (0.40–2.54)	0.979		
Age	1.00 (0.97–1.03)	0.939		
Serum IL-10 < 1.3 pg/ml	4.36 (1.67–11.42)	0.003	2.85 (1.02–7.95)	0.045
CSF WBC count >5.5/ul	3.80 (1.37–10.46)	0.010	1.92 (0.64–5.78)	0.247
Presence of CSF OCBs	0.59 (0.23–1.48)	0.259		
Baseline Gd+ MRI	8.60 (1.15–64.35)	0.036	5.87 (0.76–45.1)	0.089
MRI T2 lesions	1.00 (0.95–1.06)	0.938		
EDSS	1.16 (0.87–1.54)	0.310		

*HR, hazard ratio; CI, confidence interval; CSF, cerebrospinal fluid; WBC, white blood cell; OCBs, oligoclonal bands; Gd+, gadolinium enhancement; EDSS, Expanded Disability Status Scale*.

### Low Serum IL-10 Levels Are Associated With a Shorter Time to a Second Event

We next examined the time to relapse in patients with high and low serum IL-10 levels using Kaplan–Meier survival analysis. As depicted in Figure 3B, low serum IL-10 levels were associated with a shorter time to a second event (log-rank, *p* = 0.001).

### Correlations of Serum IL-10 Levels With Clinical and Laboratory Data

**Figure 4:**
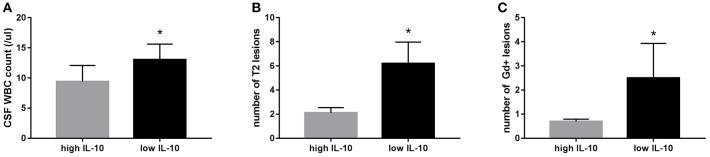
Positive correlations between serum IL-10 levels and clinical and laboratory data. Compared to the high IL-10 group, we found a significantly higher cerebrospinal fluid (CSF) white blood cells (WBC) count (*p* = 0.035) **(A)**, number of T2 lesions (*p* = 0.036) **(B)**, and number of gadolinium-enhancing (Gd+) lesions in baseline MRI (*p* = 0.049) **(C)** in the low IL-10 group. *p < 0.05 represent statistical significance in the Mann-Whitney *U*-test.

Comparing the low IL-10 and high IL-10 groups, we found significantly higher counts of CSF WBC, T2 lesions, and Gd+ lesions in baseline MRI in the low IL-10 group than those of the high IL-10 group (*p* = 0.035, *p* = 0.036, and *p* = 0.049, respectively; Figure 4). No significant differences were observed between the two groups in sex, age, CSF protein level, IgG index, or EDSS during the first-onset and last follow-up.

We also examined correlations between serum IL-10 levels and the clinical and laboratory findings (i.e., other detected cytokines levels, CSF-WBC count, protein level, and IgG index, number of T2 and Gd+ lesions, and EDSS scores during attacks and remission) using the Spearman rank correlation test. However, we were unable to find any significant correlations between serum IL-10 levels and these data.

### Conversion to MS and NMOSD

The baseline characteristics of CIS patients who converted to NMOSD (CIS-N) and MS (CIS-MS) are summarized in Table 3. The CIS-N group had more patients with longitudinally extensive transverse myelitis than the CIS-M group (62.5 vs. 0.0%, *p* = 0.026). There were no significant differences in other baseline variables between the two groups.

**Table 3:** Characteristics of three groups of clinically isolated syndromes (CIS) patients who relapsed during follow-up.

**Clinical characteristics**	**CIS-N (*n* = 8)**	**CIS-M (*n* = 7)**	**CIS-O (*n* = 5)**
Sex ratio (F/M)	7/1	4/3	2/3
Age at onset, mean (SD), years	41.3 (10.3)	36.0 (20.6)	48.2 (17.5)
**Onset location, n (%)**			
Optic nerve	0 (0.0)	2 (28.6)	0 (0.0)
Spinal cord	7 (87.5)	4 (57.1)	3 (60.0)
≥3 vertebral segments	5 (62.5)	0 (0.0)	2 (40.0)
Cerebrum	1 (12.5)	5 (71.4)	1 (20.0)
Brainstem	3 (33.3)	3 (42.9)	1 (20.0)
Number of T2 lesions, median (range)	1.5 (1–8)	5.0 (1–15)	1.0 (1–4)
Gd+ MRI, n (%)	8 (100.0)	6 (85.7)	5 (100.0)
EDSS, mean (SD)	4.9 (1.5)	4.0 (1.5)	4.1 (1.6)
**CSF analysis at onset**			
CSF WBC count, mean (SD), /ul	16.8 (21.1)	12.1 (7.8)	9.4 (7.3)
CSF protein level, mean (SD), mg/dl	41.5 (25.2)	30.4 (18.3)	40.2 (17.5)
CSF IgG index, mean (SD)	1.1 (1.5)	0.7 (0.4)	0.7 (0.3)
Positive oligoclonal bands, n (%)	3 (37.5)	6 (85.7)	4 (80.0)
Interval to second episode, mean (SD), months	14.1 (10.7)	15.3 (9.6)	21.1 (9.4)

*CIS-N, CIS patients who converted to neuromyelitis optica spectrum disorder (NMOSD); CIS-M, CIS patients who converted to multiple sclerosis (MS); CIS-O, CIS patients who relapsed without converted to MS or NMOSD; F, female; M, male; Gd+, gadolinium enhancement; EDSS, Expanded Disability Status Scale; CSF, cerebrospinal fluid; WBC, white blood cell*.

No significant differences were found in any cytokine levels between CIS patients who converted to NMOSD and those who converted to MS.

## Discussion

Prospective studies demonstrate that 60–70% of CIS patients develop a second clinically evident demyelinating event within 20 years (11, 12) and will, therefore, be diagnosed with clinically definite MS (CDMS), NMOSD, recurrent ON, relapsing myelitis, or other disorders. The identification of factors influencing the risk of relapse and conversion is important to effectively determine prognoses, thus directing early intervention strategies. This also carries the potential to further our understanding of the biological mechanisms driving these IIDDs. In this study, we examined several serum cytokines levels in CIS patients during the acute phase of their first onsets and prior to any immunomodulatory or immunosuppressive therapy. We found that serum IL-10 levels were lower in CIS patients who relapsed during follow-up and low serum IL-10 levels were a predictor of second clinically evident demyelinating events. However, among the serum cytokines detected, none could differentiate between the conversion from CIS to MS or NMOSD

As a key immunosuppressive cytokine, IL-10 is expressed by cells of the innate and the adaptive immune system, including dendritic cells (DCs), macrophages, mast cells, natural killer (NK) cells, eosinophils, neutrophils, CD4 and CD8 T cells, and B cells [reviewed in (13–15)]. IL-10 plays a crucial role in preventing inflammatory and autoimmune pathologies, by blocking the production of pro-inflammatory cytokines and hindering the capacity of myeloid cells to efficiently activate T cells (13, 14, 16, 17). Thus, IL-10 prevents excessive tissue damage caused by bacterial and viral infections as well as regulates and represses proinflammatory responses. The production of IL-10 by T cells is considered a hallmark of a subset of regulatory T (Treg) cells that inhibit or dampen immune responses (18, 19). IL-10 is associated with many autoimmune diseases. Blocking the IL-10 pathway in mice causes spontaneous development of inflammatory bowel disease (IBD) (20). Patients with genetic defects in the IL-10/IL-10R pathway develop severe early-onset colitis that can be healed by transplanting IL-10/10R-sufficient stem cells (21). A high IL-10 serum level is maintained during the early phase of remission in some human ulcerative colitis patients, whereas C-reactive protein and IL-6 serum levels return to normal during remission after an increase during the acute phase (22). This indicates that IL-10 plays an important role of maintaining normal immune tolerance of the intestine and inhibiting the autoimmune response of IBD. Similar to its functions in IBD, IL-10 also exerts protective functions during liver inflammation (23, 24).

In autoimmune diseases of the CNS, patients with MS and NMO had higher IL-10 levels in serum and CSF than those of patients with OND or healthy controls (25–27). A reduction in IL-10 producing B cells was found in relapsing-remitting MS (RRMS) patients experiencing relapse compared with patients in remission (28). MS patients infected with helminths displayed an increase in IL-10 producing CD19^+^CD1d^hi^ B cells as well as a better clinical outcome (29). In line with these results, our study also found that serum IL-10 levels were increased in patients with CIS compared to that of patients with OND. Our study also demonstrated for the first time that low serum IL-10 levels were associated with a higher risk of relapse for CIS patients and shorter time to second events. Regarding clinical correlations, a significantly higher CSF WBC count, number of T2 lesions, and number of Gd+ lesions in baseline MRI were found in the low serum IL-10 levels group. One possibility is that, as a key immunosuppressive cytokine, a decrease in IL-10 may contributes to an imbalance of pro-inflammatory and anti-inflammatory factors (30, 31). The relative predominance of proinflammatory factors leads to an increased inflammatory activity as evidenced by a higher CSF WBC count, greater number of lesions, and earlier disease reactivation (32). Another possibility is that low IL-10 is the consequence, rather than the cause, of more severe inflammation and a more aggressive disease. A low IL-10 level, high WBC count, and greater number of lesions are all indicators of a severe immune response and high risk of conversion. It is important to identify the role of IL-10 in CIS onset and the recurrence process, not only to further our understanding of its pathogenesis, but also because it has practical implications for therapy. When applied with appropriate localization, amounts, and timing, IL-10 can completely protect animals from experimental autoimmune encephalomyelitis (EAE) (33). Some of the classical disease-modifying therapies (DMTs) for MS, such as Interferon-β, Fingolimod, and Glatiramer, have an effect in increasing anti-inflammatory cytokines especially IL-10 (34, 35). Drugs are currently being developed that offer major therapeutic effects by enhancing the production of IL-10. Among these, the ABP dendrimer is most noteworthy. The efficacy of the ABP dendrimer has been evaluated in both preventive and curative therapeutic protocols in EAE mouse models and it was shown to be as efficacious as the current gold standard, Fingolimod (36, 37). These drugs may provide new therapeutic approaches for the treatment of CIS patients, but their clinical effectiveness, safety, and suitability for NMOSD patients remains to be further investigated.

In 85% of patients who later develop MS, clinical onset occurs with CIS. The term CIS usually suggests the possibility of MS. Neuromyelitis optica (NMO) is considered a severe variant of MS, and it is frequently misdiagnosed as such. The discovery of AQP4-Ab in 2004 greatly contributed to differentiating CIS from MS, revolutionizing diagnosis (38, 39). In 2015, the term “NMOSD” was adopted and it is classified into two types: AQP4-Ab-positive and AQP4-Ab-negative NMOSD (8). With improvements to our understanding of NMOSD, the conversion from CIS to NMOSD has drawn greater concern and attention (40). It has been suggested that NMO has a predilection for non-white. The proportion of NMO among IIDDs of the CNS is 1.2% in Italy, 1–2% in the USA, and 39.3% in Thailand (41, 42). The ratios of NMO/NMOSD to MS in Asia are much higher than those in Western countries. The ratios were 41% in Malays, and 63% in Chinese (41–44). However, data on the proportion and regional differences of conversion from CIS patients to NMOSD are still lacking. In the present study, of the 69 CIS patients, there were seven converted to MS and eight converted to NMOSD after a mean follow-up of 23.3 ± 8.2 months. There were more CIS patients converted to NMOSD than MS. But given the relatively small sample size and short follow-up duration in our study, larger scale prospective studies with long-term follow-up are expected to address these issues.

As CIS can convert to different IIDDs, future research is necessary for investigating factors affecting the conversion of these disorders and their predictive biomarkers. In this study, we found no significant differences in any cytokine levels between CIS patients who converted to NMOSD compared with those who converted to MS. However, in previous researches, the serum cytokine profiles of NMOSD patients were distinct from those with MS (25, 26, 45–47). This may be because the majority of these studies have analyzed a combination of treated and untreated patients, patients with first onset and recurrence, or patients with and without AQP4-Ab. Very few studies compared AQP4-Ab-positive and AQP4-Ab-negative NMOSD patients in their analysis. Our previous study showed that differences in cytokines at first-onset was more significant in AQP4-Ab-positive NMOSD patients than in AQP4-Ab-negative NMOSD patients, when compared with MS patients (27). Although the contrast found in our study may be due to a small sample size and differences in patient characteristics, whether cytokine levels change during the conversion from CIS to NMOSD or MS remains an important issue that should be examined in future research.

None of the CIS patients were positive for AQP4-Ab in serum at the first attack. This may be because AQP4-Ab-positive patients can be diagnosed with NMOSD as only one core clinical characteristic is needed, so few patients diagnosed with CIS remain. AQP4-Ab is known to be pathogenic and can be detected as early as a decade prior to the onset of the disease (48–50). Several independent studies found a higher frequency of AQP4-Ab in patients with relapsing NMO than in patients with monophasic NMO (51, 52). This implies that AQP4-Ab does not turn positive at some point after disease onset (53). Therefore, patients negative for AQP4-Ab at first-onset may remain negative when relapsed. In the present study, of the eight patients who converted to NMOSD during follow-up, serum AQP4-Abs remained negative in seven patients, while the serum antibody of one patients turned positive at relapse. The inconsistency in the present case may be because the CBA tests sensitivity ranges from 57 to 91% (54, 55), and a false negative result may occur. Another possibility is that two attacks in this case belonged to different disease processes. As a result, it is highly important to improve the sensitivities of the detection methods and to recheck AQP4-Ab in patients with recurrence.

Some limitations in our study need to be addressed. Because our patient cohort was small and derived from a single university hospital, and the follow-up duration was short, the results should be verified in larger-scale, multicenter studies with long-term follow-up. We could not adequately analyze the serial change in serum cytokine levels after first onset, and whether this change would affect the risk of recurrence and conversion could not be determined. Also the anti-MOG antibodies were not measured in our study. Further studies are expected to address these issues. In addition, it is necessary to investigate the role of the IL-10 pathway in CIS pathogenesis and therapeutic strategies.

## Conclusions

The aggregate results from this study confirm that serum IL-10 serves as a prognostic biomarker associated with the risk of relapse and the time to second events in CIS patients. We propose that serum IL-10 levels should be measured in CIS patients to identify those individuals with a high risk of relapse. This result suggests that reinforcement of the IL-10 pathway may be a promising therapeutic strategy for patients at high risk of relapse.

## Ethics Statement

The study was approved by the Ethics Committee of Beijing Tiantan Hospital Affiliated to Capital Medical University, Beijing, People's Republic of China (No. KY2015-031-02), and written informed consent was obtained from all participants.

## Author Contributions

YW, XZ, and LY conceived and designed the study. HC performed the MILLIPLEX® map human High Sensitivity Cytokine Panels tests and ELISA tests. YW, HF, and XL participated in evaluating the EDSS scores of patients and collecting blood samples. YW performed the statistical analysis. XZ and LY revised the manuscript. All authors reviewed the final manuscript.

### Conflict of Interest Statement

The authors declare that the research was conducted in the absence of any commercial or financial relationships that could be construed as a potential conflict of interest.
