# Supplementary material for: Low Serum Interleukin-10 Is an Independent Predictive Factor for the Risk of Second Event in Clinically Isolated Syndromes
Source: Front Neurol. 2019 Jun 11;10:604. doi: 10.3389/fneur.2019.00604 (PMC6579832; doi:10.3389/fneur.2019.00604)
Supplement: Supplementary file 1 [file Table_1.docx]

Supplementary Table 1. Characteristics of the clinically isolated syndromes (CIS) patients who relapsed during follow-up.

| Patient | Onset age, year/sex | First attack  site | Brain lesions | Spinal MRI lesion | Number of T2 lesions | Baseline Gd+ MRI | CSF OCB  at onset | Interval to relapse  (months) | Lesions  at last follow-up |
| --- | --- | --- | --- | --- | --- | --- | --- | --- | --- |
| 1 | 24/F | Spinal cord | No | C7-T2 | 2 | Yes | No | 35.4 | Area postrema |
| 2 | 34/F | Cerebrum, spinal cord | Deep white matter | C2-T5 | 8 | Yes | No | 4.4 | Periependymal cerebral lesions |
| 3 | 50/F | Spinal cord | No | C2-6 | 1 | Yes | No | 5.5 | LETM (C4-7) |
| 4 | 34/F | Brainstem, spinal cord | Dorsal medulla | C1 | 3 | Yes | Yes | 17.9 | Hypothalamus, thalamus and periependymal surfaces of the third ventricle |
| 5 | 50/F | Brainstem | Midbrain and pons | No | 1 | Yes | Yes | 9.1 | LETM (T5-8), medulla oblongata |
| 6 | 53/F | Spinal cord | No | C3-6 | 1 | Yes | No | 23.0 | Pons to C1 |
| 7 | 47/F | Spinal cord | No | C2-T4 | 1 | Yes | Yes | 8.0 | ON+LETM (C3-T3) |
| 8 | 38/M | Brainstem, spinal cord | Medulla oblongata | C1 | 2 | Yes | No | 9.6 | Extensive subcortical white matter lesion, LETM (C2-6, T6-10) |
| 9 | 14/F | Cerebrum | Deep white matter + periventricular | No | 15 | Yes | Yes | 28.0 | Periventricular, cerebellum |
| 10 | 51/M | Brainstem, spinal cord | Medulla oblongata | T8 | 2 | Yes | Yes | 25.4 | Pons |
| 11 | 15/F | Cerebrum | Periventricular + deep white matter | No | 14 | Yes | No | 8.1 | Periventricular, juxtacortical |
| 12 | 57/M | Cerebrum | Juxtacortical | No | 1 | Yes | Yes | 4.7 | Medulla oblongata |
| 13 | 18/F | ON, spinal cord | No | C2,C4-5, T4, T11-12 | 5 | Yes | Yes | 9.7 | Medulla oblongata, C3 |
| 14 | 36/F | Brain, spinal cord | Deep white matter +medulla oblongata | C3 | 7 | No | Yes | 22.3 | L3-4 |
| 15 | 61/M | ON, brain, spinal cord | Deep white matter, Pons | C2-3 | 4 | Yes | Yes | 8.7 | Medulla oblongata, C2 |
| 16 | 47/F | LETM | No | T9-12 | 1 | Yes | Yes | 31.5 | T4-6 |
| 17 | 32/M | Spinal cord | No | C2-3 | 1 | Yes | Yes | 27.8 | C3 |
| 18 | 35/M | Spinal cord | No | C1-3 | 1 | Yes | No | 7.1 | C3 |
| 19 | 76/M | Brainstem | Pons, medulla oblongata | No | 2 | Yes | Yes | 19.0 | Pons |
| 20 | 51/F | Cerebrum | Deep white matter (frontal lobe) | No | 4 | Yes | Yes | 19.9 | Tumefactive demyelination (temporal lobe) |

Patients 1-8 converted to neuromyelitis optica spectrum disorder; the serum aquaporin-4 antibody of patient 8 was negative at first-onset but turned positive at relapse; patients 9-15 converted to MS; patients 16-18 were diagnosed with relapsing myelitis; patient 19 with relapsing brainstem encephalitis, and patient 20 with tumefactive demyelination. F, female; M, male; Gd+: gadolinium enhancement; CSF, cerebrospinal fluid; OCBs: oligoclonal bands; LETM, longitudinally extensive transverse myelitis; ON, optic neuritis.
