# Supplementary material for: Low Serum Interleukin-10 Is an Independent Predictive Factor for the Risk of Second Event in Clinically Isolated Syndromes
Source: Front Neurol. 2019 Jun 11;10:604. doi: 10.3389/fneur.2019.00604 (PMC6579832; doi:10.3389/fneur.2019.00604)
Supplement: Supplementary file 2 [file Data_Sheet_1.docx]

Supplementary Figure 1. Receiver operator characteristics (ROC) curves of serum IL-10 levels and cerebrospinal fluid (CSF) white blood cells (WBC) count.


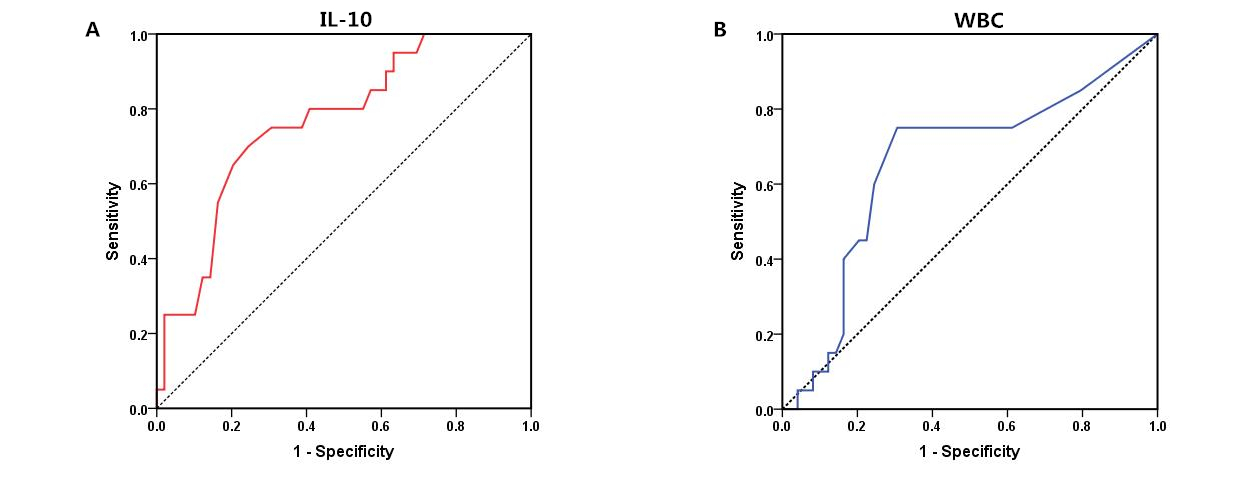


A. The areas under the ROC curve (AUC) of serum IL-10 were significant with respect to relapse (AUC = 0.77; 95% confidence interval [CI] 0.65–0.89; *p* = 0.001).

B. The AUC of the CSF WBC count were significant with respect to relapse (AUC = 0.66; 95% CI 0.51–0.81; *p* =0.042).
